# Gossypiboma-induced ileal obstruction following cholecystectomy: A case report and literature review

**DOI:** 10.1016/j.ijscr.2025.111138

**Published:** 2025-03-11

**Authors:** Mohammad Asef Adelyar, Mahmoud Tavakkoli, Ghulam Saki Hassani, Muska Anwary, Ramin Saadaat, Maiwand Anwari

**Affiliations:** a Department of Abdominal Surgery and Abdominal Oncological Surgery, Aliabad Teaching Hospital, Kabul University of Medical Sciences, Kabul, Afghanistan; b Department of Urology, Imam Reza Hospital, Mashhad University of Medical Sciences, Mashhad, Iran; c Department of Urology, Aliabad Teaching Hospital, Kabul University of Medical Sciences, Kabul, Afghanistan; d Department of Orthodontics, Stomatology Teaching Hospital, Kabul University of Medical Sciences, Kabul, Afghanistan; e Department of Histopathology, French Medical Institute for Children, Kabul, Afghanistan; f Department of General Surgery, Jamhuriat Hospital, Kabul, Afghanistan

**Keywords:** Gossypiboma, Surgical complications, Intestinal obstruction, Transmural migration, Retained surgical sponge, Patient safety

## Abstract

**Introduction:**

Gossypiboma, the retention of surgical material in the body post-surgery, is a rare but serious complication often underreported due to legal and reputational concerns. It commonly occurs after abdominal surgeries, with presentations ranging from abscess formation to intestinal obstruction.

**Presentation of case:**

An 18-year-old girl presented with abdominal pain and distention, along with vomiting for six weeks after cholecystectomy. Initial misdiagnoses as pancreatitis delayed appropriate treatment. Exploratory laparotomy revealed an intra-abdominal gossypiboma, which had transmigrated into the ileum, causing obstruction. Surgical removal of the retained pack and ileal resection with primary anastomosis were performed, leading to a successful recovery.

**Discussion:**

The incidence of gossypiboma varies widely and depends on surgical practices. Transmural migration into the gastrointestinal tract, as seen in this case, is rare and potentially life-threatening. Accurate sponge counts, use of radio-opaque materials, and adherence to safety protocols are crucial preventive measures. Imaging modalities aid diagnosis, but surgical exploration remains definitive in uncertain cases.

**Conclusion:**

This case highlights the critical need for awareness, accurate surgical protocols, and vigilance in postoperative management to prevent and manage gossypibomas. Early recognition and intervention can mitigate complications and improve patient outcomes.

## Highlights

• Gossypiboma is a rare but serious postoperative complication with significant medical and legal implications.
• A young girl developed intestinal obstruction due to the transmural migration of a retained surgical sponge.
• Misdiagnosis initially delayed treatment, highlighting the challenges in identifying gossypibomas.
• Surgical removal and ileal resection resolved the condition, emphasizing the importance of timely intervention.
• Preventive measures, including accurate sponge counts, radiopaque markers, and adherence to surgical protocols, are crucial.

## 1 Introduction

The term “gossypiboma” is derived from the Latin word gossypium (cotton) and the Swahili word boma (place of concealment). It is also referred to as textiloma, originating from the Latin textilis (weave) and the Greek oma (disease, tumor, or swelling) [1]. Other terms used interchangeably include cottonoid, cottonballoma, and gauzeoma [2].

Gossypibomas are reported in various surgical specialties, including abdominal, thoracic, cardiovascular, orthopedic, and neurosurgical procedures. This condition describes a retained textile matrix surrounded by a foreign-body reaction, with gauze, surgical sponges, and dressings being the most common retained items following abdominal surgeries. The reported incidence of gossypibomas varies and is likely underreported due to medicolegal concerns and asymptomatic cases [1]. The incidence of retained items is about 1 in every 10,000 to 1500 laparotomies, depending on the type of surgery, whether elective or emergency. The cotton sponge is the most commonly retained object [3].

The clinical manifestations of gossypibomas differ based on their location and the type of inflammatory response. Acute cases typically present as abscesses and fistulas, while chronic cases manifest as encapsulated masses with nonspecific symptoms. Surgical removal, either through endoscopy, laparoscopy, or open surgery, is the standard treatment to prevent complications, including a mortality rate of 11 %–35 % [1].

Transmural migration of an intra-abdominal gossypiboma is a rare yet serious complication. The most commonly affected organs or cavities in this migration include the stomach, ileum, small bowel, colon, bladder, and vagina. Other reported sites of involvement include the pericardium, nose, urethra, and diaphragm [4]. In rare instances, a gossypiboma may transmigrate into the gastrointestinal lumen without causing any defects, enabling it to exit the body through the intestines. Otherwise, laparoscopic or open surgery would be required for its removal [5]. Herein, we report a case of gossypiboma transmigrate into the gastrointestinal lumen, which is a rare complication of gossypiboma. Our work has been reported in concordance with the SCARE criteria [6].

## 2 Case presentation

An 18-year-old girl was admitted to the surgical department with abdominal pain, distention, nausea, and vomiting lasting six days. She reported a weight loss of 3 kg and a history of cholecystectomy performed approximately 40 days ago.

**Fig. 1:**
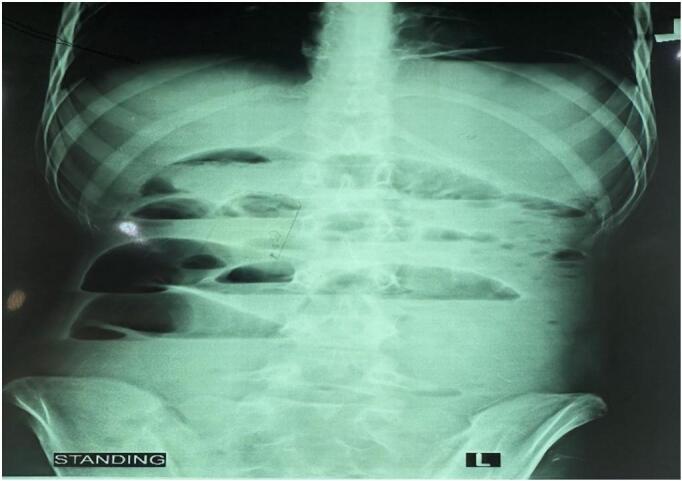
Preoperative abdominal x ray shows air-fluid levels.

On examination, her vital signs were stable, but significant abdominal distention and tenderness were noted. A firm, sausage-shaped mass was palpable in the right lower quadrant. Blood tests revealed leukocytosis (whole blood count of 16,000/mm^3^), mild anemia (hemoglobin of 10 g/dL), and borderline elevated urea and creatinine levels. An abdominal X-ray showed dilated small bowel loops with multiple air-fluid levels (Fig. 1).

Prior to this admission, the surgical team that performed her cholecystectomy had evaluated her at another hospital. They suspected pancreatitis due to gallstones based on elevated serum amylase levels 700 U/L, and treated the patient using pancreatitis management protocols, along with fluid therapy, antibiotics and antiemetics for five days. Although her symptoms initially improved, but worsened shortly after discharge.

After being admitted to the hospital and undergoing physical examinations and laboratory tests, a Computed Tomography (CT) scan was recommended as a further diagnostic tool to identify the cause of the illness. However, it could not be performed due to financial constraints of the patient. Consequently, the surgical team decided to carry out an exploratory laparotomy.

Intraoperative findings included an abscess collection and adhesions over the first part of the duodenum. After drainage of abscess and adhesiolysis, a 3 cm perforation was identified in the first part of the duodenum which was repaired by two-plans and omentopexy also was done.

**Fig. 2:**
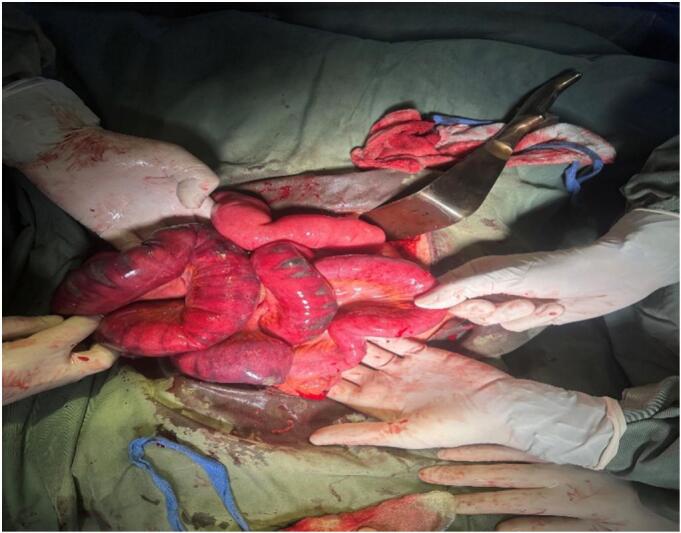
An intra-operative image displaying dark, patchy areas of gangrene in the small bowel.

**Fig. 3:**
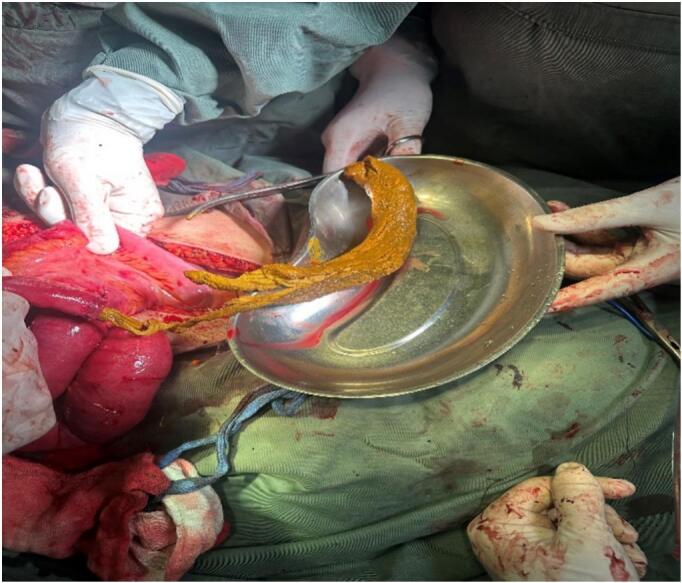
Intra-operative image, showing the removal of a surgical pack from the terminal ileum.

Examination of the intestine revealed multiple gangrenous lesions in the ileum and a distended terminal ileum caused by a foreign body (Fig. 2). At first, the surgical team suspected that the ileal obstruction might have been caused by ascariasis. The obstruction was immovable with milking. After resection of approximately 1 m of the ileum, a 40 × 30 cm retained abdominal pack was extracted from terminal ilium, followed by primary ileo-ileal anastomosis (Fig. 3). tube drains were applied into the peritoneal cavity.

The patient recovered well postoperatively and was discharged in stable condition after twelve days.

## 3 Discussion

The earliest documented case of a retained surgical item, known as gossypiboma, was reported by Wilson in 1884 [2].

The diagnosis of gossypiboma can lead to serious medicolegal issues between the patient and surgeon, often resulting in significant repercussions for the surgeon. These may include widespread negative media coverage, personal humiliation, emotional trauma, damage to professional reputation, and the possibility of legal action. Although rare, the transmural migration of retained gauzed has been documented and can result in complications such as fistula formation with nearby organs or intestinal obstruction. This migration is thought to occur due to pressure from necrosis in the bowel wall, triggered by an encapsulated segment of the intestine. The encapsulation arises from the peritoneum's inflammatory response to presence of the foreign object [2].

The small bowel, particularly the ileum, is the most frequently affected area of the gastrointestinal tract, though there have been a few reported cases involving the colon and stomach. Transmural migration is most commonly liked to cholecystectomy, followed by cesarean sections and hysterectomies [7].

Interestingly, up to 88 % of reported cases of retained surgical materials occurred despite a correct surgical count being documented before wound closure. The use of radioopaque sponge has been associated with a reduced incidence of retained surgical items, and their use should be recommended [2].

Key risk factors for gossypiboma include emergency surgeries, unexpected intraoperative changes, high body mass index, rushed sponge counts, prolonged procedures, patient instability, and surgical inexperience [5,8]. When a radiological marker is absent, diagnosing a retained sponge through imaging become challenging, as it can mimic radiologic features of conditions such as hematomas, neoplasms, granulomatous reactions, abscesses, cystic masses, calcifications, or air bubbles. Contrast-enhanced Magnetic resonance imaging (MRI) is considered the most reliable imaging method for identifying granulomas caused by retained gauze [8,9].

Silva CS et al. reported a case of complete migration of a retained surgical sponge from the abdominal cavity into the ileum in a 24-year-old woman. This occurred four months after a cesarean section and presented with symptoms such as diffuse colicky abdominal pain, nausea, vomiting, and constipation. The patient underwent an ileotomy with subsequent anastomosis, and no fistulas or open intestinal wall defects were observed [8]. Prevention remains the most effective approach. To minimize the risk of complications and legal issues, the surgical team must collaborate to ensure accurate counting of all tools and gauze before concluding the procedure. Additionally, intraoperative radiologic screening and routine radiographs for high-risk patients at discharge are recommended [8].

Several protocols have been proposed to prevent gossypibomas in operating rooms, but no universal consensus exists, and each method has its limitations. Sponges should be individually separated, counted out loud, and visually verified by both the scrub nurse and the circulating nurse during the counting process. In the event of a discrepancy, the entire surgical team must take necessary actions to locate the missing item. A persistent mismatch between the initial and final sponge counts increases the risk of a retained foreign body by 100-fold [3].

A staff radiologist should review the film with clear instructions that its purpose is to “rule out a retained foreign body. Before anesthesia is reversed, the radiologist must relay the results of the review to the surgeon and ensure that the imaging covers the entire surgical field, such as the abdomen, pelvis, or other relevant body cavities [10].

The time to diagnose a retained surgical sponge has been reported to vary widely, from as short as one day [11] to as long as 40 years [12]. In the immediate postoperative period, the most common clinical indication of a retained sponge is a surgical site infection. While sponges are sterile, any contamination can allow bacteria to thrive within the sponge's internal spaces, which are inaccessible to the immune system. This can result in wound infections, abscesses, fistulas, or even sepsis. Acute intra-abdominal sepsis, a severe complication associated with high morbidity, occurs in approximately 10–15 % of cases involving retained surgical sponges [3]. Diagnosing gossypibomas can be difficult and requires a thorough clinical assessment, including a detailed medical history and physical examination. Radiological imaging methods such as X-rays, CT scans, and MRIs can provide useful insights but may not always conclusively identify gossypibomas. In situations where suspicion remains high despite inconclusive imaging results, exploratory surgery is necessary to establish a definitive diagnosis and promptly remove the retained surgical material [4].

While non-surgical methods, such as percutaneous radiological retrieval of foreign bodies, have been suggested as potential alternatives, they are not always effective and carry the risk of additional complications. The American College of Surgeons emphasizes the importance of maintaining a focused surgical environment, which requires operating rooms to remain quiet and free from distractions [4]. Gossypiboma progresses through four stages: foreign-body reaction, secondary infection, mass formation, and remodeling. Pathological reactions to the foreign body can take one of two forms: a fibrinous response, leading to adhesions and encapsulation, or an exudative response, resulting in abscess formation [13].

Basic precautions, such as educating staff, utilizing the World Health Organization (WHO) Surgical Safety Checklist, marking sponges with identifiable tags, and performing multiple perioperative counts of sponges and materials, can effectively lower the risk of gossypiboma [14].

## 4 Conclusion

Intra-abdominal gossypiboma is a rare but serious postoperative complication that can lead to gastrointestinal symptoms and delayed diagnosis. It should be considered in patients with a history of abdominal surgery and unexplained symptoms. Preventive measures, such as strict adherence to surgical protocols, sponge counts, and staff education, are essential to reduce risk and legal implications. In this case, a rare transmigration of a surgical sponge into the duodenum caused an intestinal obstruction at the ileocecal junction.

## Consent

Written informed consent was obtained from the patient for publication of this case report and accompanying images. A copy of the written consent is available for review by the Editor-in-Chief of this journal on request.

## Ethical approval

The manuscript has got an ethical review exemption from the Ethical Review Committee (ERC) of our institution, as case reports are exempted from review according to the institutional ethical review committee's policy.

## Guarantor

The corresponding author is the guarantor of article.

## Patient perspective

The patient expressed that publishing and sharing this case with other healthcare professionals would be valuable in enhancing understanding, preventing gossypiboma, and ensuring accurate diagnosis of such rare cases, which require specific treatment and follow-up.

## Research registration number

Not applicable.

## Provenance and peer review

Not commissioned; externally peer-reviewed.

## Funding

No financial support was provided for this study.

## Author contribution

MAA convinced the idea. MAA operated the patient. MAA, MaiA and MA were involved in literature review and drafted the manuscript. RS and MAA helped to collect clinical and follow-up data of the cases. RS, MT and GSH provided histopathological images and description. MA and MT participated in reviewing the drafted manuscript. MAA participated with the corresponding, editing the drafted manuscript as per journal policy, and submission of the article. All authors read and approved the final manuscript.

## Conflict of interest statement

The authors declare that their work is not funded by any institution, organ, or government and they have no financial support.
